# New Insight into Food-Grade Emulsions: Candelilla Wax-Based Oleogels as an Internal Phase of Novel Vegan Creams

**DOI:** 10.3390/foods13050729

**Published:** 2024-02-28

**Authors:** Iwona Szymanska, Anna Zbikowska, Sylwia Onacik-Gür

**Affiliations:** 1 Department of Food Technology and Assessment, Institute of Food Science, Warsaw University of Life Sciences–SGGW, 159C Nowoursynowska Street, 02-776 Warsaw, Poland; anna_zbikowska@sggw.edu.pl; 2 Department of Meat and Fat Technology, Prof. Waclaw Dabrowski Institute of Agriculture and Food Biotechnology—State Research Institute, 36 Rakowiecka Street, 02-532 Warsaw, Poland; sylwia.onacik-gur@ibprs.pl

**Keywords:** oleogels, candelilla wax, O/W emulsions, vegan food, cream analogues

## Abstract

Cream-type emulsions containing candelilla wax-based oleogels (EC) were analyzed for their physicochemical properties compared to palm oil-based creams (EP). The microstructure, rheological behavior, stability, and color of the creams were determined by means of non-invasive and invasive techniques. All the formulations exhibited similar color parameters in CIEL*a*b* space, unimodal-like size distribution of lipid particles, and shear-thinning properties. Oleogel-based formulations were characterized by higher viscosity (consistency index: 172–305 mPa·s, macroscopic viscosity index: 2.19–3.08 × 10^−5^ nm^−2^) and elasticity (elasticity index: 1.09–1.45 × 10^−3^ nm^−2^), as well as greater resistance to centrifugal force compared to EP. Creams with 3, 4, or 5% wax (EC3–5) showed the lowest polydispersity indexes (*PDI*: 0.80–0.85) 24 h after production and the lowest instability indexes after environmental temperature changes (heating at 90 °C, or freeze–thaw cycle). EC5 had particularly high microstructural stability. In turn, candelilla wax content ≥ 6% *w*/*w* accelerated the destabilization processes of the cream-type emulsions due to disintegration of the interfacial layer by larger lipid crystals. It was found that candelilla wax-based lipids had great potential for use as palm oil substitutes in the development of novel vegan cream analogues.

## 1. Introduction

O/W emulsions are excellent matrices in the design of food with increased nutritional value. The physical stability of an emulsion is determined by its quantitative and qualitative composition, e.g., volume fraction and particle size distribution of the lipid phase [1], chemical composition of lipids [2], type and concentration of emulsifier [3], and thickeners [4]. Finding a compromise between stability and nutritional value of food remains a major challenge. Therefore, it is necessary to conduct research on the modification of technology and reformulation of the composition, leading to the improvement of quality and functionality of food emulsion products [5]. Physicochemical properties of food products that are O/W emulsions (e.g., mayonnaises, dressings, sauces, milk, creams, yoghurts, drinks, desserts) largely depend on the characteristics of both the aqueous phase and the lipid phase [2]. However, the emulsion science lacks research on the simultaneous nutritional and structural modification of lipids constituting the inner phase of O/W-type systems.

Consumers suffer from deficiencies of essential n-3 polyunsaturated fatty acids (PUFAs, e.g., α-linolenic acid), which are biologically active compounds having a beneficial effect on the heart, brain, and eyesight [6]. At the same time, exaggerated consumption of saturated fatty acids (SFAs) increases the risk of cardiovascular diseases, which are the main cause of death globally [7,8]. The excessive supply of SFAs in the diet results, among others, from the wide availability of popular food products containing semi-solid or solid fats [9]. However, this applies not only to full-fat dairy products and high-fat meat products [10], but also to their plant-based substitutes (with coconut oil and palm oil) [11,12]. Palm oil is the most frequently used plant oil in the food industry worldwide. Its popularity results from it having the highest production yield, resulting in high availability and quite low price. However, controversies over deforestation and labor exploitation causes consumers to often avoid products with palm oil. Therefore, there are nutritional, health, environmental, and social aspects that support replacing palm oil in food production [13,14].

From a technological point of view, replacing solid fat (with a high content of SFAs) with oil (with a high content of PUFAs) often leads to a decrease in product stability [15]. In order to obtain lipids with the desired properties, various methods of their structuring are used [16]. One of the methods that can bring a number of benefits is enzymatic interesterification, but it is also a long-term process [17] and can generate high production costs due to the use of enzymes [18]. Therefore, alternative methods are still being explored. In recent years, scientific research on oleogelation has received great interest [19]. Oleogelation is defined as a method of structuring low-viscosity, non-polar fluids using various structuring agents [20]. It enables obtaining systems with a semi-solid or solid consistency at room temperature (21 ± 2 °C), with a small mass fraction (up to several %) of the structuring substance (oleogelator). This process does not cause changes in the chemical composition or distribution of fatty acids in the TAG structure [21]. It has been proven that oleogelation can not only stabilize the oils physically [22,23] and/or oxidatively [24,25], but also delay the process of lipid digestion, which allows for the controlled release of the bioactive compounds contained therein [26,27].

The properties of oleogels depend mainly on the types of base oil and structuring substance and the oleogelation conditions [28,29]. The oleogels obtained from unrefined oils, containing a lot of accompanying substances, are characterized by weaker three-dimensional structures and lower oil-binding capacity [30,31,32] than refined oil-based oleogels [29,32]. The vegetable waxes (e.g., carnauba, candelilla, rice, sunflower) are very effective structuring agents for oils. However, the varied composition, structure, and properties of waxes determine their role in food matrices [33,34]. The oleogelator that works well in many different research projects is candelilla wax [21,24,32,33]. This wax has received GRAS status (Generally Recognized as Safe) and has been approved as a permitted food additive (E 902) [35,36]. According to the Codex Alimentarius [37], candelilla wax can be used as a carrier, emulsifier, glazing agent, or thickener, but in compliance with good manufacturing practices.

One of the richest sources of n-3 PUFAs are linseed and rapeseed oils [38]. The preliminary studies [39] have shown that mixing these refined oils in a 1:1 (*w*/*w*) ratio gives a mixture (RO-LO) with approx. 30% α-linolenic acid in the pool of fatty acids, as well as showing relatively high oxidative stability. It was also found that such a well-composed mixture of oils can be a great basis for microstructural modification of lipids and O/W-type emulsions. Other previous research [39] allowed to conclude that the use of candelilla wax concentrations as low as 3% *w*/*w* enabled the obtaining of an oleogel (based on an RO–LO mixture) with greater physical stability than palm oil. At the same time, numerous researchers [40,41,42,43] highlighted the suitability of this wax for developing food-grade emulsions. There is an increase in evidence of the technological benefits of using oleogels in the design of food products, including bakery and confectionery products [44], spreadable fats [45], ice cream [46], chocolate products [47], and meat products [48]. Nevertheless, due to changes in dietary trends towards limiting the consumption of animal products, there is also a need for research to develop vegan alternatives to dairy products, including cheese, yogurts, and creams [49,50,51]. Therefore, the objective of this study was to evaluate the influence of candelilla wax-based oleogels, obtained with a rapeseed and linseed oil mixture, on the physicochemical properties of O/W cream-type emulsions. Moreover, this study aimed to compare oleogel-based creams, containing various candelilla wax contents to their palm oil-based counterpart and assess the potential of using these structured lipids to develop vegan cream analogues.

## 2. Materials and Methods

### 2.1. Materials

Refined rapeseed oil and refined palm oil were purchased from Bunge Polska sp. z o.o. (Kruszwica, Poland); refined linseed oil from Poltrade, Ltd. (Kraków, Poland); and organic soybean drink from a local market (Warsaw, Poland). Food-grade candelilla wax was kindly supplied by IMCD Poland, Ltd. (Warsaw, Poland). Sodium benzoate (>99% purity) was provided by Chempur (Piekary Śląskie, Poland).

### 2.2. Preparation of Oleogels

The oleogels (100 g) were prepared according to our previous research [39]. Firstly, 3–7% candelilla wax (*w*/*w*) was dispersed in a mixture of refined rapeseed and linseed oils (1:1 *w*/*w*) by heating for 10 min at 80 ± 1 °C in a water bath (AJL Electronic, Krakow, Poland) and then sonication for 10 s (26 kHz, 72 W, 100% pulse, 100% amplitude) using the ultrasound homogenizer UP200St (Hielscher Ultrasonics GmbH, Oderstr., Germany), equipped with the titanium sonotrode S26d7. The clear, homogeneous mixture was statically cooled in a thermostatic cabinet (ST 2/2+, POL-EKO APARATURA, Wodzislaw Slaski, Poland) for 24 h at 20 ± 1 °C, until the proper structure was formed. The oleogels were obtained in three repetitions.

### 2.3. Preparation of Cream-Type Emulsions

Cream-type O/W (30/70 *w*/*w*) emulsions (100 g) were prepared according to the procedure in our preliminary research [38] with slight modifications. Both an aqueous phase (soybean drink containing 2.6% *w*/*w* protein) and a lipid phase (palm oil or oleogel) were preheated up to 55 °C and immediately homogenized by using the ultrasound homogenizer UP200St (Hielscher Ultrasonics GmbH, Oderstr., Germany) with a frequency of 26 kHz. The following parameters were used: 100% pulse, 80% amplitude, 15 mm immersion of the sonotrode in the central part of the beaker (200 mL capacity). The homogenization time of 2.5 min (τ) was determined on the basis of the results of technological tests and preliminary tests, taking into account the increasing temperature of the samples (for τ = 2.5 min: energy density: 69.1 ± 0.4 J∙g^–1^, max. temperature: 61.0 ± 0.3 °C). Sodium benzoate of 0.15% (*w*/*w*) was added to the aqueous phase as an antibacterial agent. The obtained samples, i.e., EP—cream-type emulsions based on palm oil—and EC3/4/5/6/7—cream-type emulsions based on oleogel with 3, 4, 5, 6 or 7% *w*/*w* candelilla wax—were stored for 24 h at 20 ± 1 °C, in order to undergo microstructural stabilization before the analyses. The creams were prepared in three repetitions.

### 2.4. Methods

#### 2.4.1. Color Parameters of Cream-Type Emulsions

The color parameters of creams were determined by means of the reflectance method using a CR-5 stationary colorimeter (Konica Minolta, Tokyo, Japan) in the CIEL*a*b* system (D65 illuminant, 10° observer). The samples were transferred to a transparent Petri dish and evenly distributed (layer of the sample: 5 cm diameter, 2 cm height), the device was calibrated against the white standard, and the measurements were conducted with the Specular Component Excluded (SCE) at 22 ± 1 °C. The measurements were performed at a temperature of 22 ± 1 °C.

L* is lightness, and a* and b* are color coordinates: where L* = 0 is black, L* = 100 is white, +a* is the red direction, −a* is the green direction, +b* is the yellow direction, and −b* is the blue direction. The total color difference (∆*E*) of the emulsion, compared to the reference sample, was calculated according to the equation [52]:

(1) $$∆E=\sqrt{{\left({L^{*}}_{1}-{L^{*}}_{0}\right)}^{2}+{\left({a^{*}}_{1}-{a^{*}}_{0}\right)}^{2}+{\left({b^{*}}_{1}-{b^{*}}_{0}\right)}^{2}}$$

where: L*_0_, a*_0_, and b*_0_ are the color parameters of the comparative sample (EP), L*_1_, a*_1_, and b*_1_ are the color parameters of the experimental sample (EC3-7).

The whiteness index (*WI*), referring to the similarity of the sample whiteness (0–100) to the standard whiteness (100), was calculated according to the equation [53]:

(2) $$WI=100-\sqrt{{\left(100-L^{*}\right)}^{2}+{a^{*}}^{2}+{b^{*}}^{2}}$$

#### 2.4.2. Microstructure of Cream-Type Emulsions

The microstructure of cream-type emulsions was characterized by means of optical microscopy. This characterization was carried out in terms of lipid particle size and distribution [54]. Dispersed particles were imaged with an optical microscope (100 TP, Delta Optical, Minsk Mazowiecki, Poland), connected to a digital camera (DLT-Cam PRO 2MP), and converted to size measurements using compatible software (DLT-Cam Viewer 3.7). The microstructure of the creams was analyzed 24 h after emulsification. A droplet of the sample was placed on the glass slide, covered with a cover slip, and then immediately observed at 600× magnification (in a bright field). A dozen or so micrographs of each sample were taken, and then three of them were selected for measurements. Approximately 1000 dispersed particles were measured for each sample. The results were computed using Microsoft Excel (Microsoft^®^ Excel, 2010). The lipid particles were grouped into classes, and then the number-weighted particle size distributions (PSD), indicating the cumulative and differential percentage of the number of lipid particles of a certain size, were determined. The mean size (number length mean: D[1,0]) of lipid particles were obtained from arithmetic values of all the lipid particle sizes in a given sample. Moreover, the polydispersity index (*PDI*), which is a dimensionless measure of the width of the size distribution ranging from 0 to 1, was calculated according to the equation [55]:

(3) $$PDI=\frac{\left(DV90-DV10\right)}{DV50}$$

where *DV90*, *DV50*, and *DV10* represent the cumulative number-weighted particle size distribution of 10%, 50%, and 90% particles, respectively.

#### 2.4.3. Rheological Properties of Cream-Type Emulsions

The rheological properties of cream-type emulsions were characterized by both non-invasive and invasive methods. The viscoelastic behavior of the samples in a native state was determined by a Rheolaser™ Master instrument (Formulaction, Toulouse, France) using Multi-Speckle Diffusing-Wave Spectroscopy (MS-DWS), according to the patented algorithm [56]. The samples are penetrated by a coherent laser beam (λ = 650 nm) at a height of 24 mm, and the backscattered light pattern is collected by a multi-pixel detector (CCD camera, 27 Hz). The measurements are recorded as a mean square displacement (*MSD*) plot on a log scale via RheoSoft Master 1.4.0.1 software [57]. The “quick characterization” test was set for 5 min at a temperature of 20 ± 1 °C. The following microrheological parameter were computed: elasticity index–*EI* (nm^−2^) (reciprocal of the *MSD* value on the plateau of the curve in the middle decorrelation time), solid–liquid balance coefficient–*SLB* (nm^2^) (*MSD* value in the middle decorrelation time), macroscopic viscosity index–*MVI* (nm^−2^) (reciprocal of the *MSD* value in the long decorrelation time). The above microrheological parameters are proportional to the viscoelastic parameters measured using an oscillatory rheometer over a wide range of frequencies. *EI* is directly proportional to the elasticity modulus *G’* expressed in Pa, while *SLB* corresponds to the dimensionless *tan*Δ = *G*″*/G*′ [57,58]. In turn, the *MVI* corresponds to the apparent viscosity at zero shear in mPa∙s [59].

The creams were also subjected to shear forces using a Brookfield DV3T rotational viscometer (Brookfield Engineering Laboratories, Inc., Middleboro, MA, USA). The viscosity test was performed at an increasing shear rate (every 10 rpm, from 10 to 250 rpm) under controlled conditions using a cylindrical adapter connected to a water bath (20 ± 1 °C). The viscometer was equipped with a DIN-86 spindle [60]. Viscosity curves (changes in apparent viscosity as a function of shear rate) and flow curves (changes in shear stress as a function of shear rate) on a logarithmic scale were determined. Due to the highest values of determination coefficients (*R*^2^ ≥ 0.95), indicating strong relationship between variables, the flow curves were finally described by the Ostwald de Waele (power law) model:

(4) $$\tau =K\cdot \gamma^{n}$$

where *τ*—shear stress (Pa), *K*—consistency index (mPa∙s), *γ*—shear rate (s^−1^), *n*—flow index (-).

Rheocalc T 2.1 software was used for this purpose. Furthermore, coefficients of variation of apparent viscosity (*Vc*), expressed in %, were calculated to estimate the shear sensitivity of the samples [34].

#### 2.4.4. Stability of Cream-Type Emulsions against Environmental Stresses

The stability of cream-type emulsions was assessed in terms of lipid particle size (Section 2.4) and resistance to centrifugal force. The second one was analyzed using a LUMiSizer 612 (LUM GmbH, Berlin, Germany), equipped with cutting-edge STEP-Technology^®^, which enables the obtainment of Space- and Time-resolved Extinction Profiles over the entire height of the centrifuged sample. The method is referred to as accelerated Centrifugal Stability Analysis (CSA). The transmitted light (near infrared light: *λ* = 870 nm) is detected by more than 2000 detectors on the CCD-line camera. The intensity of light transmission is recorded as a transmission profile (“fingerprints”) and recomputed using SepView 6.0 software [61,62]. The samples of cream-type emulsions (0.5 mL) were transferred to test tubes, then centrifuged at a constant temperature (20 ± 1 °C) for 50 min at 4000 rpm. Transmission curves were captured every 30 s. The measure of the degree of sample destabilization was the dimensionless total instability index, ranging from 0 to 1. The evolution of the instability index value as a function of centrifugation time was presented in a line chart generated using Microsoft Excel 2021 software.

The stability of the emulsions was tested 24 h after production and after subjecting the samples to the following environmental factors:

- Heating samples (10 g) were heated at a temperature of 90 ± 1 °C for 30 min in a water bath (AJL Electronics, Krakow, Poland), then cooled in a thermostatic cabinet at a temperature of 20 ± 1 °C for 3 h prior to stability analyses [63].
- freeze-thawing samples (10 g) were frozen at a temperature of −18 ± 1 °C for 24 h, then thawed in a thermostatic cabinet at a temperature of 20 ± 1 °C for 3 h prior to stability analyses [64].
- changes in ionic strength–samples (10 g) were dispersed in previously prepared NaCl solutions (0.05 M, 0.10 M, and 0.15 M) or with distilled water (0 M) in a 1:1 (*v/v*) ratio using a magnetic stirrer (600 rpm, 10 min), then incubated in a thermostat cabinet at a temperature of 20 ± 1 °C for 24 h prior to stability analyses [65].
- changes in pH value–samples (10 g) were dispersed in buffer solutions with a pH of 5, 7, or 9 in a 1:1 ratio (*v*/*v*) using a magnetic stirrer (600 rpm, 10 min), then incubated in a thermostatic cabinet at a temperature of 20 ± 1 °C for 24 h prior to stability analyses [66].

#### 2.4.5. Statistical Analysis

Statistical analyses of the results were performed using Statistica 13.3 software (TIBCO Software Inc., Palo Alto, CA, USA). A significance level of *α* was set to 0.05 (significant differences between variables: *p*-value < 0.05).

In order to compare the mean values of the parameters (dependent variables), a one-way analysis of variance (ANOVA) test was performed (grouping variable: cream type). The following tests were used to verify the hypotheses: Shapiro–Wilk (normal distribution), Levene’s and Brown–Forsythe (equality of means), and sigma-constrained parameterization (equality of variance). Homogeneous groups were determined using Tukey’s post hoc (HSD) test [67].

The strength of the correlation between variables was determined using the Pearson correlation test. Correlation coefficients (*ρ*) and *p*-values were determined. The following correlation assessment criterion was adopted: very strong (|*ρ*| = 1.0), strong (0.8 ≤ |*ρ*| < 1.0), moderate (0.5 ≤ |*ρ*| < 0.8), weak (0.1 ≤ |*ρ*| < 0.5), very weak (0.01 ≤ |*ρ*| < 0.10). A negative value of the correlation coefficient means an inversely proportional relationship between the variables (negative correlation), and a positive value means a directly proportional relationship (positive correlation) [68].

## 3. Results and Discussion

### 3.1. Color Parameters of Cream-Type Emulsions

Due to the fact that color is the first food feature assessed by the consumer, it was examined how the modification of the lipid phase influenced the color of cream-type emulsions. The color of the emulsion depends on its ability to scatter and absorb light. This is related to the composition and structure of the emulsions as well as destabilization processes [3,69]. Interestingly, the physical properties of O/W emulsions are contingent upon the characteristics of the lipids used in the internal phase [2].

**Figure 1:**
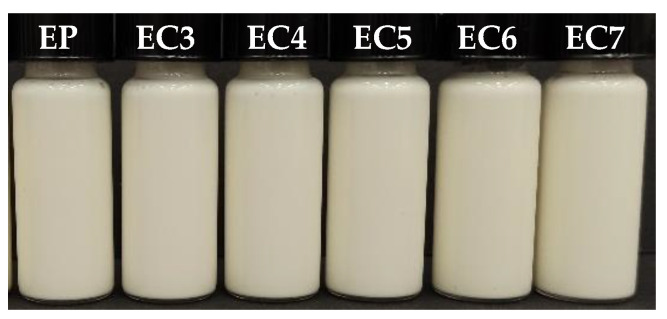
Visual appearance of cream-type emulsions (24 h after production). EP–cream-type emulsions based on palm oil; EC3/4/5/6/7–cream-type emulsions based on oleogel with 3, 4, 5, 6, or 7% *w*/*w* candelilla wax.

The visual appearance of the cream-type emulsions is shown in Figure 1 and described by color coordinates in Table 1. All tested formulations reached equally high brightness (*p*-value > 0.05); the values of the L* coordinate exceeded 90. High L* values of milk products and milk-like counterparts are connected with the efficiency of ultrasound treatment and the reduction of lipid particles to smaller sizes [70].

Moreover, the type of lipid phase did not affect the differences in the a* values (close to 0). In contrast, EC3-5 were characterized by significantly lower b* values compared to EP. The mean values of the b* coordinate, which is indicative of the yellowness, varied between 8.61 and 9.26 (Table 1). Chudy et al. [52] emphasized that a positive b* value is a typical feature of the natural color of dairy products.

On the basis of the color components L*, a*, and b*, the whiteness indexes (*WI*) and the total color differences in relation to palm oil-based emulsion (Δ*E*_EP_) were determined (Table 1). The oleogel-based creams were characterized by a slightly higher value of *WI* compared to EP. The cause of this observation may be related to the differences in the structure of lipid particles (due to the presence of wax at the interface), causing distinctions in light absorption. A similar tendency was noticed by Javidi et al. [70], who studied the color of low-fat emulsions with waxy cornstarch nanocrystals used as a fat substitute.

The mean values of Δ*E*_EP_, which ranged from 0.27 to 0.70, indicated no visible differences in color between EP and oleogel-based emulsions. As demonstrated by Chudy et al. [52], at Δ*E* < 1.0, color differences may be imperceptible even to an experienced observer. Thus, it can be assumed that such modification of the lipid phase does not cause changes in the appearance of the potential product, which is the first feature that the consumer evaluates when making a purchasing decision [71,72].

Although the examined formulations do not differ in terms of macroscopic appearance, they could show dissimilar internal characteristics. Therefore, in the following part of this research, the emulsions were subjected to microstructural, rheological, and stability analyses.

### 3.2. Microstructure of Cream-Type Emulsions

**Figure 2:**
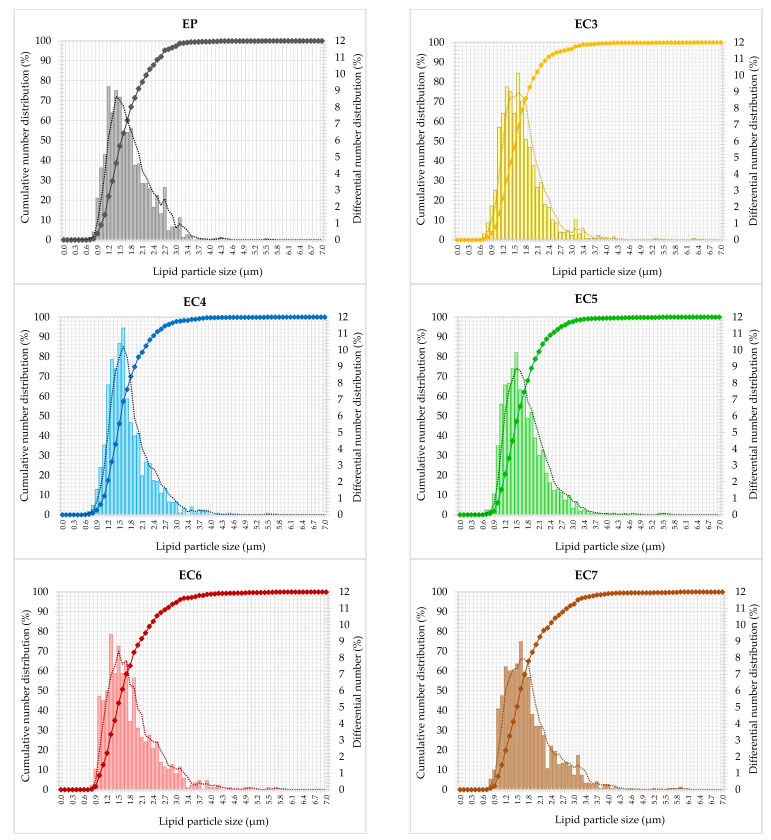
Cumulative (scatter plots with lines) and differential (vertical bars) number distribution curves of cream-type emulsions. For denomination of cream-type emulsions, see Figure 1.

The size of dispersed particles is one of the basic indicators of the stability of O/W-type emulsions [3]. Therefore, the cumulative and differential % number distributions (Figure 2) show the individual patterns of lipid particle size span in each emulsion tested. The cumulative particle size distribution, i.e., the sum of the differential distributions, appeared as single S-shaped curves with quite steep positive slopes in the middle of the particle size range. The dispersed particles of all the formulations ranged from 0.6 to 6.3 µm in size. The particle size distributions seem to be unimodal with a major contribution of the lipid particles with the sizes less than about 1.6 µm. Differences between emulsions were visible for classes of larger particles, where the slope of the cumulative curves decreased, especially in samples with the highest wax concentration (EC6 and EC7). These systems contain more numerous classes of larger particles (especially in range from 2.1 to 4.0 µm) compared to other emulsified samples. Although all the differential particle size distributions were relatively narrow, they do not have perfectly smooth shape (Figure 2). This means that the vegan cream-type emulsions have not strong unimodality, irrespective of lipid type incorporated. On the other hand, EC4 and EC5 distinguish from the other samples. Their differential size distributions were shifted to the left, i.e., to lower sizes of lipid particles (Figure 2). Smaller particles of internal phase usually mean a lower probability of early destabilization of the O/W emulsion [73]. As shown in Table 2, the number-weighted mean size of lipid particles in oleogel-based creams with 3–5% candelilla wax (CW) was not significantly different from the palm oil-based emulsion. The use of higher concentrations of wax, i.e., 6–7% for the structuring of oils resulted in an increase in the value of this parameter (Figure S1). However, the mean particle size is a parameter that is not sufficient to comprehensively conclude about emulsion stability. Therefore, based on the cumulative number distributions of lipid particle size, the *D*-values (*DV10*, *DV50,* and *DV90*) were determined, and polydispersity indexes (*PDI*) were calculated. The lower value of the polydispersity index, the greater the similarity of the emulsion to the monodisperse system, i.e., showing greater physical stability [3,74,75]. The lowest values of polydispersity indexes (0.80–0.85) were obtained for oleogel-based creams with 3%, 4%, and 5% wax content (EC3–EC5). In turn, creams with other structured lipids (containing 6–7% of CW in a lipid phase) showed significantly higher values of this parameter in comparison with EP (0.92) (Table 2).

**Figure 3:**
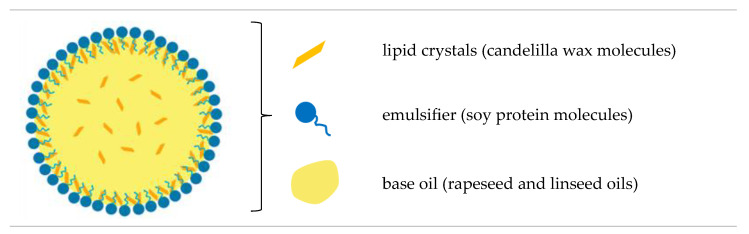
Illustration of the structure of a single lipid particle in an oleogel-based cream-type emulsion.

In general, the characteristics of the creams were influenced mainly by the high efficiency of emulsification via ultrasonic homogenization. Whereas the microstructural diversity of vegan creams resulted mainly from the composition and the phenomenon of lipid recrystallization during static cooling. The presence of numerous, large lipid crystals in the emulsion favors the joining of lipid particles into larger aggregates [3,76]. As we have shown in another study, palm oil was characterized by the largest lipid crystals compared to candelilla wax-based oleogels [39]. Higher values of polydispersity indexes for EC6 and EC7 could result from a larger number of crystals compared to other structured lipids. Therefore, it can be concluded that the concentration of candelilla wax in the lipid phase of the emulsion at a level of ≥6% *w*/*w* hindered the dispersion of structured lipids in the aqueous phase. Munk et al. [77] proved that after the emulsification of oleogels with the aqueous phase at a temperature above the melting point of these lipids, the original structure of the oleogels is not rebuilt, but most of the molecules of the structuring substance migrate to the lipid-water interface, where they form a shell of lipid particles (Figure 3).

Based on the above microstructural characteristics of cream-type emulsions, it can be concluded that the concentration of candelilla wax (CW) had a significant effect on the size of dispersed particles and their distribution. The use of 3–5% CW allowed to obtain the O/W emulsions with similar microstructural properties to the palm oil-based emulsion. Higher CW concentrations contributed to presumably worse properties. Nevertheless, this could be explained by further research involving rheological analyses.

### 3.3. Rheological Characteristics of Cream-Type Emulsions

**Figure 4:**
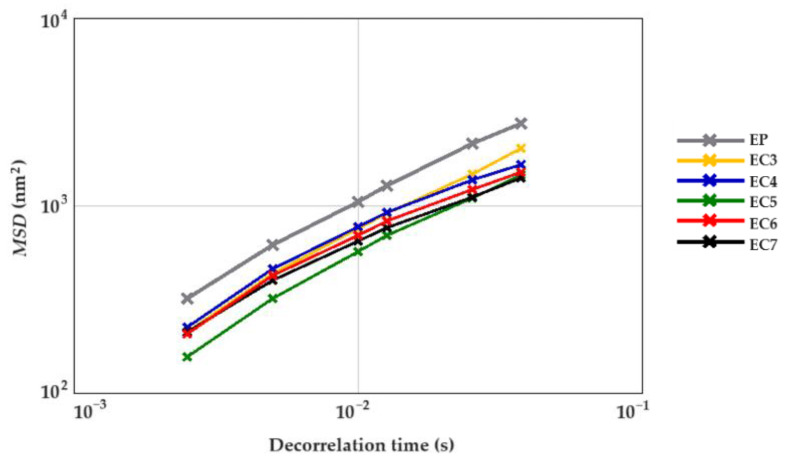
Mean Square Displacement (*MSD*) curves of cream-type emulsions (24 h after production). For denomination of cream-type emulsions, see Figure 1.

In order to comprehensively assess the rheological behavior of cream-type emulsions, both non-invasive and invasive techniques were used. Firstly, the samples were examined in their natural (undamaged) state. For this purpose, the formulations were subjected to the micro-scale analysis by means of the non-invasive diffusing wave spectroscopy method, which is based on the migration of particles due to Brownian motion [59]. The evolution of Mean Square Displacement (*MSD*) in the function of decorrelation time was determined for each sample. It allows to characterize the viscous and viscoelastic properties of the creams. The linear increase in the *MSD* value informs about the free movement of particles, which is characteristic of a “purely viscous” system [78,79]. Figure 4 shows the *MSD* curves of all cream-type emulsions 24 h after production. The curves resemble a straight line. Thus, it was concluded that all the creams exhibited liquid behavior. Similar conclusions were reached by Xu et al. [79], analyzing the microrheological properties of O/W emulsions stabilized with whey protein isolate with the addition of 0.1%, 0.2%, or 0.3% *w*/*w* or without the addition of the linseed gum.

The palm oil-based cream (EP) did not differ from the oleogel-based cream with 3% candelilla wax (EC3) in terms of the average elasticity index (*EI*). Other oleogel-based creams (EC4–7) were characterized by higher EI values compared to the above-mentioned samples. There was no significant relationship between the CW concentration in the lipid phase and the average elasticity index of the cream-type emulsions. The EC5 was characterized by the greatest microstructural elasticity, i.e., about two times higher than EP (Table 3). It was found that the cream-type emulsion with 5% CW in the dispersed phase had the highest degree of packing of molecules in the internal structure as well as greater physical stability. According to Degrand et al. [56] and Medronho et al. [80], the increase in the elasticity of the system may be associated with a reduction in the size of lipid particles and, at the same time, an increase in the number of these particles, limiting the free movement in the structure.

The mean values of the coefficient of solid–liquid balance (*SLB*) of tested creams were above 0.5 (Table 3), which confirms that the liquid-like behavior of these samples was dominant [59]. The lowest *SLB* values were obtained via EC6 and EC7 (Table 3). Thus, it was noticed that the concentration of candelilla wax had a significant influence on the microrheological properties of oleogel-based creams. Moreover, a strong positive correlation was found between the mean *SLB* values and the flow index *n* (*ρ* = 0.90; *p*-value = 0.0064). Therefore, it can be stated that the higher the *SLB* value, the greater the fluidity of the cream-type emulsion. According to Wang et al. [81], a high *SLB* value, which indicates high mobility of dispersed particles, may be related to sedimentation and phase separation of the emulsion.

The type of lipid phase also affected the macroscopic viscosity (at zero shear) of the obtained creams. The oleogel-based emulsion with the lowest wax content (3% *w*/*w*) was characterized by an approximately 1.5-fold higher *MVI* value (2.19 × 10^−5^ nm^−2^), compared to EP. In addition, an increase in the candelilla wax content in the lipid phase resulted in an increase in the viscosity of the cream-type emulsions. The mean *MVI* value for EC7 was about 67% greater compared with EC3 (Table 3).

**Figure 5:**
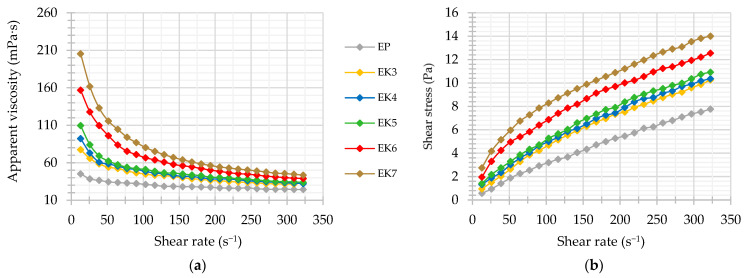
Apparent viscosity (**a**) and flow (**b**) curves of cream-type emulsions. For denomination of cream-type emulsions, see Figure 1.

After investigating the microrheological properties of the samples using the non-invasive method, they were also subjected to shear forces. As a result, flow curves and viscosity curves were obtained. Increasing the shear rate resulted in a decrease in apparent viscosity (Figure 5a) and an increase in shear stress (Figure 5b). However, these changes were not directly proportional. The greatest decrease in viscosity was observed at low shear rates, when the particle flow was turbulent. As flow rates increased, viscosity decreased as shear broke larger particles into smaller particles until the structure became ordered, and at the highest shear rates, the viscosity stabilized at a relatively constant level. Such behavior is typical for non-Newtonian shear-thinning (or pseudo-plastic) fluids. In addition, the shape and course of the flow curves, intersecting the origin of the coordinate system (Figure 5b), prove that examined formulations did not have a yield point [82]. The shear thinning phenomenon is associated with a change in the orientation of particles (including proteins) in the direction of the induced flow in order to reduce the friction force. At the same time, weaker intermolecular bonds (e.g., hydrogen, hydrophobic) may be broken and the internal structure of the emulsion irreversibly disturbed [83]. Additionally, the presence of crystals in the lipid phase of the emulsion is responsible for crystalline particle–particle interactions, which are also favored by their irregular shapes. However, these interactions are relatively unstable and susceptible to mechanical forces [84]. To express numerically the degree of change in apparent viscosity under shear, coefficients of variation were determined for each emulsion variant. High values of coefficients of variation of apparent viscosity *Vc* (>20%) indicated a significant sensitivity of the creams to shear forces. The value of *Vc* was higher for the systems with higher initial apparent viscosity, e.g., *Vc* for EC7 it was about twice as high as for EC3 (Table 4). A similar relationship was also found by Quintana-Martinez et al. [85], who analyzed the rheological properties of O/W emulsions with different contents of guar gum and lecithin.

Therefore, to expand the rheological properties of cream-type emulsions, the experimental data were described with a power law model with a high degree of fit (*R*^2^ ≥ 0.95). The overall consistency coefficients (*K*) and flow indexes (*n*) were determined. All the creams were characterized by the value of the flow index *n* < 1.0, confirming a non-Newtonian shear-thinning characteristics. Compared to the oleogel-based creams, EP was characterized by a greater fluidity (Table 4). Zhang et al. [66] also noted that the oleogel-in-water emulsions showed higher viscosity than traditional emulsions. In turn, the rheological behavior of the oleogel-based creams was sensitive to a change in candelilla wax concentration. When CW content increased, a significant increase in the consistency coefficient and decrease in the flow index were observed (Table 4). It was mainly due to the increase in viscosity of the lipid phase as a result of the oleogelation [38]. As reported by Elik et al. [86] a lower value of *n* means a higher degree of shear thinning. Thus, a higher wax concentration could have increased the pseudoplasticity of the emulsion. According to the interpretation proposed by Goyal et al. [87], EP belongs to emulsions with low pseudoplasticity (0.77–0.80), while EC6 and EC7 be-long to emulsions with high pseudoplasticity (0.56–0.59).

Both non-destructive and destructive analyses allowed for a quite complex characterization and comparison of the rheological properties of cream-type emulsions. The results of non-destructive test showed that the use of candelilla wax for structuring the lipid phase of the O/W emulsion promoted strong intermolecular interactions. In turn, as observed in destructive test, these bonds are sensitive to shear forces, causing significant changes in the macroscopic viscosity of the systems. Therefore, the next step in the research was to analyze the stability of these formulations.

### 3.4. Physical Stability of Cream-Type Emulsions

The obtained cream-type emulsions were considered as potential vegan analogues of sweet cream for soups or sauces. Such products are characterized by different pH levels and ionic strengths and can be subjected to heat treatment before consumption. Due to the probable application, the stability of the samples was analyzed 24 h after production and after being subjected to environmental stress.

#### 3.4.1. Stability of the Creams 24 h after Production

**Figure 6:**
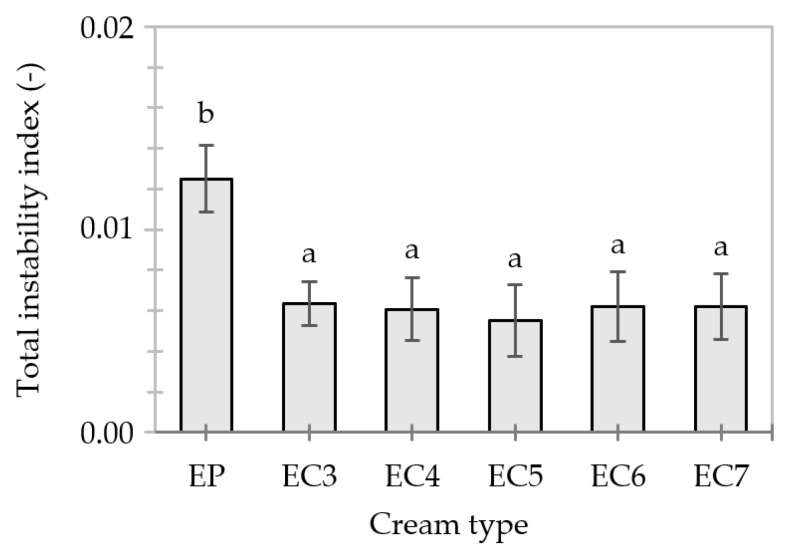
Total instability indexes of cream-type emulsions 24 h after production. For denomination of cream-type emulsions, see Figure 1. Different letters indicate significant differences (grouping variable: cream type), *p*-value < 0.05.

The transmission profiles of “fresh” creams show that the flotation of lipid particles (i.e., creaming) was initiated (Figure S2). EP exhibited about a 2-fold lower value of the total instability index than EC3–7 (Figure 6). The greater resistance of EC6–7 to centrifugal force could be related to the specific structure of lipid particles stiffened with a layer of candelilla wax and, at the same time, the higher consistency coefficient of these dispersions under shearing compared to EP (Table 4). Thus, the effect of replacing the popular palm oil with unconventional lipids, obtained through the oleogelation, on the stability of cream-type emulsions was highlighted.

Moreover, the oleogel-based creams did not differ in terms of mean values of the total instability index (*p*-value < 0.05)–Figure 6. Thus, the stability of these creams 24 h after production was not influenced by candelilla wax content (3–7%). In turn, Guo et al. [88] showed that the instability of an oleogel-in-water (20/80) emulsion can occur with an increase in rice wax concentration in the dispersed phase to about 4%, due to excessive crystallization and the growth of intra-particle wax crystals. It should be noted that rice wax crystals are much longer (20–50 µm) [40,89] than candelilla wax crystals (8–16 µm) [39]. Although the length of the wax crystals greater than 10 µm is a desirable feature for gel formation, it is not conducive to the preparation of stable O/W-type emulsions.

#### 3.4.2. Stability of Cream-Type Emulsions after Heating

Transmission profiles of the heated (90 °C, 30 min) creams (Figure S3) resembled the profiles of “fresh” emulsions (Figure S2), proving the occurrence of similar destabilization mechanisms. However, the higher level of light transmission in the case of heated creams suggests less stability of these samples, which was confirmed by the increase in the values of the total instability index. At a temperature of 90 °C, all lipid crystals present in the emulsions melted, which increased the mobility of the molecules. Oleogel-based creams showed greater thermal stability than EP. This could be related to the higher melting point of oleogels, which was also noticed by Zhang et al. [66]. In addition, protein–lipid junctions at the interface could increase the resistance of the emulsions to high temperatures.

**Figure 7:**
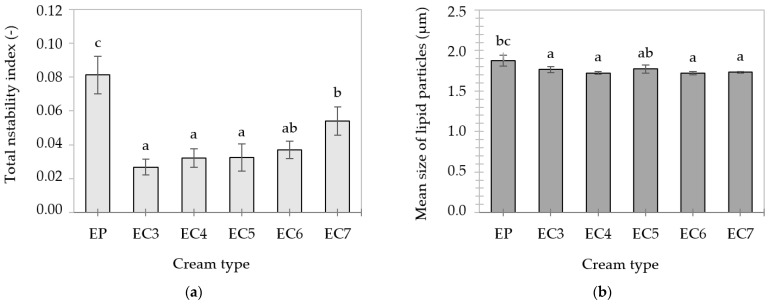
Total instability indexes (**a**) and mean size of lipid particles (**b**) of cream-type emulsions after heating at 90 °C for 30 min. For denomination of cream-type emulsions, see Figure 1. Different letters indicate significant differences (grouping variable: cream type), *p*-value < 0.05.

The concentration of CW in the lipid phase of heated creams did not have a statistically significant effect on the centrifugal stability of EC3–7 (Figure 7a). It was observed that the mean size of lipid particles in the creams after heating was larger in the case of EP and EC3 (Figure 7b), compared to their “fresh” counterparts (Table 2). In addition to the phenomenon of flotation (creaming) of lipid particles, their partial aggregation may have occurred.

Modifications of protein properties may contribute to changes in the stability of O/W emulsions. According to Keerati-u-rai & Corredig [90], heating induces denaturation and the formation of protein aggregates on the surface of dispersed particles. According to Dapueto et al. [51], the lipid particle size in O/W emulsions (30/70) increases with the degree of thermal denaturation of proteins.

#### 3.4.3. Stability of Cream-Type Emulsions after Freeze-Thawing

During the centrifugation of previously frozen and then thawed creams, there was a significant increase in the intensity of light transmission (Figure S4), hence these samples showed relatively high instability. The uneven course of the profile curves indicates a different migration rate of particles (larger ones migrate faster than smaller ones), which may result from the polydisperse distribution of emulsions [63].

**Figure 8:**
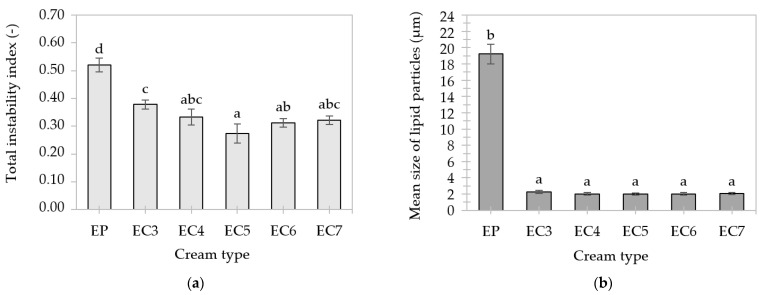
Total instability indexes (**a**) and mean size of lipid particles (**b**) of cream-type emulsions after freeze–thaw cycle. For denomination of cream-type emulsions, see Figure 1. Different letters indicate significant differences (grouping variable: cream type), *p*-value < 0.05.

The palm oil-based cream (EP) was characterized by the greatest instability, because the first curve (marked with a black arrow in Figure S4) indicated a high level of transmission. The macroscopic appearance of EP after thawing resembled a curd (marked with a yellow arrow in Figure S4), which means that the cycle of freezing and thawing caused a significant decrease in its stability (due to coagulation). As a result, the total instability index increased to a value above 50 (Figure 8a) and the mean size of lipid particles to about 19.2 μm (Figure 8b). This could be due to the presence of very large lipid crystals in the dispersed phase of EP, which “protrude” beyond the interfacial layer of lipid particles, increasing the risk of their joining into larger clusters. When freezing O/W emulsions, the lipid phase with a high melting point crystallizes faster than the aqueous phase, which also promotes the aggregation of dispersed particles [91]. It was observed that the use of high-melting lipids to obtain the cream-type emulsions prevented the phenomenon of complete coalescence (“emulsion collapse”) after thawing, even in the case of the least stable oleogel-based cream (EC3).

Oleogel-based creams did not differ in the mean size of lipid particles (about 2.0–2.2 μm), as shown in Figure 8b. The CW particles could strengthen the interfacial layer and contribute to greater adsorption of soy proteins. Scientific research confirms that complexes of proteins with certain substances (e.g., phospholipids, polysaccharides) increase emulsion stability compared to systems containing only proteins [81,92]. The smallest decrease in stability occurred in the oleogel-based cream with a 5% concentration of candelilla wax (EC5), i.e., the value of the instability index did not exceed 30 (Figure 8a).

#### 3.4.4. Stability of Cream-Type Emulsions at Various pH Values

Changing the pH of the environment affected the stability of the cream-type emulsions. The creams dispersed in a solution with a pH = 5, i.e., near the isoelectric point (*pI*) of soy proteins, turned out to be the least stable (Table 5). There was a decrease in the solubility of proteins and an acceleration of the creaming phenomenon. However, the shape of the transmission profiles (regular course of the curves, similar degree of transmission increase, and the “sharp front” of the profile, marked with a black arrow in Figure S5) suggests that all the tested creams retained a unimodal particle size distribution. Moreover, all the samples were relatively stable in an environment with pH equal to 7 and 9 (Figure S5) and did not show statistically significant differences in the mean size of lipid particles (Table 5). Zhang et al. [66] confirmed the high stability of soy proteins at pH > 6. In turn, Mao et al. [65] obtained the most stable oleogel-based (soybean oil structured with monoacylglycerols) emulsions at a pH equal to 7 or 9 (pH range: from 3 to 11).

#### 3.4.5. Stability of Cream-Type Emulsions at Various Ionic Strengths

The cream-type emulsions were also subjected to different ionic strengths, and then their physical stability was analyzed. Based on the obtained transmission profiles (Figure S6), it was found that in all the creams, the flotation of lipid particles (creaming) progressed relatively intensively. Regardless of the concentration of the NaCl solution used, profiles of “fingerprints” with a shape characteristic for systems with polydisperse distributions were obtained (Figure S6). Both the transmission profiles (Figure S6) and the values of the total instability index (Table 6) indicate a decrease in the resistance of the emulsions to centrifugal force under the influence of an increase in the ionic strength (sodium chloride concentration) of the environment. In addition, no statistically significant differences were observed between the mean values of the total instability index for the emulsions dispersed in a solution with the same concentration of NaCl. However, differences were noted between the samples (the same type of cream) subjected to different ionic strengths. EC6 and EC7 were characterized by about a 2-fold higher value of the total instability index at 0.15 M NaCl compared to 0 M NaCl. In the case of EP, these differences reached about 1.5 times (Table 6) higher value. A significant effect of increasing the concentration of NaCl on the acceleration of destabilization of oleogel-based emulsions was also shown by Mao et al. [65].

Changes in the ionic strength of the environment increased the mean size of lipid particles in all the creams but did not exceed a size of 2.5 μm. The highest value of this parameter was found for EC7 (about 2.26 μm) at 0.15 M NaCl (Table 6). At a given ionic strength of the environment (up to 0.15 M NaCl), the electrostatic repulsion of the lipid particles of the emulsion was strong enough to prevent the demulsification process of the systems. The use of a NaCl solution concentration above 0.15 M could lead to intensive flocculation of dispersed particles, as in the research of Taha et al. [63]. It is assumed that the lipid crystals present in the internal phase of the emulsion may reduce its sensitivity to high concentrations of sodium chloride. In addition, a significant increase in the mean size of dispersed particles, under the influence of increasing the ionic strength, is a frequent phenomenon in the emulsions obtained with non-crystallized oils [66]. According to Munk and Andersen [76], substances that structure hydrophobic systems (e.g., monoacylglycerols), which are present in the lipid phase of the emulsion, can form a crystal network that immobilizes oil droplets and prevents oil separation.

Taking into account the influence of changes in various external factors (temperature, ionic strength, pH level) on the stability of the emulsions enabled the imitation of the conditions of their potential use and allowed the identification of differences and similarities. A surprising result was the high stability of the oleogel-based emulsions in freeze–thaw cycle, which is rare in the case of O/W emulsions.

### 3.5. Strengths and Limitations of the Research

As this study is the continuation of a previous research [38,39], its strengths were that the influence of candelilla wax concentration on the physicochemical properties of cream-type emulsions could be observed and compared to emulsion with palm oil in a more comprehensive way. Based on the results collected and analyzed so far, it can be concluded that the oleogel-based emulsions showed promising potential for use as vegan creams. This is particularly important due to the lack of research focusing on the utilization of the oleogelation method in this area of food production.

The limitation of this study could be that it was carried out using one type of protein (soy) and specific time–temperature conditions. The use of plant protein derived from other raw materials and time/temperature changes could affect the properties of the dispersions. Consumer preferences change over time; therefore it is also difficult to predict the consumer’s perception of candelilla wax presence in the product composition. Moreover, this research included only the systems 24 h after production and did not include either aging tests as a function of storage time or chemical (e.g., oxidative) analyses. Additionally, the emulsions were prepared in laboratory conditions; therefore, in the next stages of research, their production should be transferred to at least a semi-technical scale for a specific food application, e.g., sauce, and then its sensory evaluation could be conducted.

## 4. Conclusions

In this study, the influence of candelilla wax-based oleogels on the physicochemical properties of cream-type emulsions was evaluated. Oleogel-based creams exhibited greater stability compared to palm oil-based creams. This was presumably caused by the migration of the wax molecules to the interfacial layer of the emulsion, thereby enhancing the structural stability of dispersed lipid particles. In addition, the concentration of candelilla wax was a significant factor determining the properties of oleogel-based creams. The wax content exceeding 6% *w*/*w* accelerated the emulsion destabilization processes due to the formation of larger lipid crystals, promoting the aggregation of dispersed particles. Nevertheless, the creams with structured lipids were relatively resistant to temperature changes, especially after freeze-thawing.

Oleogel-based emulsions containing 3–5% *w*/*w* candelilla wax exhibited the most desirable features in terms of the analyzed variables. However, the obtained results did not allow for a clear indication of the optimal concentration of the wax. Therefore, it is expected to explore changes in the physical and oxidative stability of these novel vegan creams as a function of storage time.

## Figures and Tables

**Table 1:** Color parameters of cream-type emulsions (mean value ± standard deviation).

Cream Type	L* (-)	a* (-)	b* (-)	*WI* (-)	Δ*E*_EP_ (-)
EP	90.9 ± 0.1 b	−0.7 ± 0.1 a	9.3 ± 0.1 c	87.0 ± 0.1 a	-
EC3	90.7 ± 0.2 ab	−0.6 ± 0.0 a	8.6 ± 0.1 a	87.3 ± 0.1 b	0.7 ± 0.1
EC4	90.9 ± 0.1 b	−0.6 ± 0.1 a	8.8 ± 0.2 ab	87.3 ± 0.1 b	0.4 ± 0.1
EC5	90.8 ± 0.1 b	−0.6 ± 0.1 a	8.9 ± 0.1 ab	87.2 ± 0.1 ab	0.4 ± 0.1
EC6	90.9 ± 0.1 b	−0.6 ± 0.0 a	9.0 ± 0.2 bc	87.2 ± 0.1 ab	0.3 ± 0.2
EC7	90.9 ± 0.1 b	−0.6 ± 0.1 a	9.1 ± 0.1 bc	87.1 ± 0.2 ab	0.3 ± 0.2

For denomination of cream-type emulsions, see Figure 1. L*, a*, b*–basic color coordinates (-), *WI—*whiteness index (-), Δ*E*_EP_—total color difference, compared to palm oil-based cream (-). Mean values ± standard deviation with different lower-case letters in the same column are significantly different (grouping variable: cream type), *p*-value < 0.05.

**Table 2:** Mean size of lipid particles and polydispersity indexes of cream-type emulsions (mean value ± standard deviation).

Cream Type	Mean Size of Lipid Particles (μm)	Polydispersity Index (-)
EP	1.67 ± 0.03 a	0.92 ± 0.02 b
EC3	1.62 ± 0.02 a	0.80 ± 0.01 a
EC4	1.69 ± 0.03 ab	0.85 ± 0.02 a
EC5	1.65 ± 0.03 a	0.83 ± 0.02 a
EC6	1.74 ± 0.02 bc	0.99 ± 0.03 c
EC7	1.75 ± 0.02 bc	1.01 ± 0.01 c

For denomination of cream-type emulsions, see Figure 1. Mean values ± standard deviation with different lower-case letters in the same column are significantly different (grouping variable: cream type), *p*-value < 0.05.

**Table 3:** Microrheological parameters of cream-type emulsions (mean value ± standard deviation).

Cream Type	*EI* × 10^3^ (nm^−2^)	*MVI* × 10^5^ (nm^−2^)	*SLB* (nm^2^)
EP	0.84 ± 0.08 a	1.37 ± 0.04 a	0.84 ± 0.01 c
EC3	1.09 ± 0.07 b	2.19 ± 0.08 b	0.80 ± 0.02 bc
EC4	1.29 ± 0.03 c	2.52 ± 0.08 bc	0.79 ± 0.02 b
EC5	1.62 ± 0.07 d	2.71 ± 0.26 c	0.78 ± 0.01 b
EC6	1.34 ± 0.05 c	2.85 ± 0.11 cd	0.70 ± 0.02 a
EC7	1.45 ± 0.05 c	3.08 ± 0.12 d	0.66 ± 0.02 a

For denomination of cream-type emulsions, see Figure 1. *EI*—elasticity index (nm^−2^); *MVI*—macroscopic viscosity index (nm^−2^), *SLB*—solid-liquid balance (nm^2^). Mean values ± standard deviation with different lower-case letters in the same column are significantly different (grouping variable: cream type), *p*-value < 0.05.

**Table 4:** Rheological parameters of cream-type emulsions under shearing (mean value ± standard deviation).

Cream Type	*K* (mPa·s)	*n* (-)	*R* ^2^	*Vc* (%)
EP	90.81 ± 4.64 a	0.77 ± 0.01 d	0.96	23.57 ± 1.07 a
EC3	172.77 ± 4.90 b	0.72 ± 0.01 c	0.98	27.04 ± 0.73 b
EC4	221.40 ± 3.92 c	0.67 ± 0.01 b	0.97	30.51 ± 1.50 c
EC5	254.16 ± 4.66 d	0.65 ± 0.01 b	0.95	35.37 ± 1.03 d
EC6	293.87 ± 3.92 e	0.59 ± 0.01 a	0.95	46.21 ± 0.91 e
EC7	304.83 ± 5.23 e	0.56 ± 0.01 a	0.96	52.50 ± 0.87 f

For denomination of cream-type emulsions, see Figure 1. *K*—consistency index (mPa∙s); *n*—flow index (-); *R*^2^—coefficient of determination (-); *Vc*—coefficient of variation of apparent viscosity (%). Mean values ± standard deviation with different lower-case letters in the same column are significantly different (grouping variable: cream type), *p*-value < 0.05.

**Table 5:** Total instability indexes and mean size of lipid particles of cream-type emulsions, dispersed in solutions with a pH of 5, 7, or 9 (1:1) (mean value ± standard deviation).

Cream Type	pH = 5	pH = 7	pH = 9
	Instability index (-)		
EP	0.25 ± 0.02 abB	0.02 ± 0.01 abA	0.01 ± 0.00 aA
EC3	0.22 ± 0.01 aB	0.02 ± 0.00 abA	0.01 ± 0.00 aA
EC4	0.21 ± 0.02 aB	0.01 ± 0.00 abA	0.01 ± 0.00 aA
EC5	0.25 ± 0.02 abB	0.01 ± 0.00 abA	0.01 ± 0.00 aA
EC6	0.29 ± 0.00 bB	0.01 ± 0.00 abA	0.01 ± 0.00 aA
EC7	0.29 ± 0.00 bB	0.01 ± 0.00 aA	0.01 ± 0.00 aA
	Mean size of lipid particles (µm)		
EP	1.7 ± 0.0 aA	1.7 ± 0.1 aA	1.7 ± 0.1 aA
EC3	1.7 ± 0.0 aA	1.6 ± 0.0 aA	1.7 ± 0.0 aA
EC4	1.7 ± 0.0 aA	1.7 ± 0.0 aA	1.7 ± 0.0 aA
EC5	1.7 ± 0.0 aA	1.7 ± 0.0 aA	1.7 ± 0.0 aA
EC6	1.7 ± 0.0 aA	1.7 ± 0.0 aA	1.7 ± 0.1 abA
EC7	1.8 ± 0.0 aA	1.8 ± 0.0 aA	1.8 ± 0.0 aA

For denomination of cream-type emulsions, see Figure 1. Mean values ± standard deviation of the parameter with different lower-case letters in the same column are significantly different (grouping variable: cream type), *p*-value < 0.05; mean values ± standard deviation of the parameter with different uppercase letters in the same row are significantly different (grouping variable: pH value), *p*-value < 0.05.

**Table 6:** Total instability indexes and mean size of lipid particles of cream-type emulsions, dispersed in solutions with a NaCl concentrations of 0 M, 0.05 M, 0.1 M or 0.15 M (1:1) (mean value ± standard deviation).

Cream Type	0 M NaCl	0.05 M NaCl	0.10 M NaCl	0.15 M NaCl
	Instability index (-)			
EP	0.15 ± 0.01 abA	0.19 ± 0.01 bcB	0.19 ± 0.00 aB	0.22 ± 0.01 aB
EC3	0.12 ± 0.01 aA	0.16 ± 0.01 aAB	0.19 ± 0.01 aB	0.20 ± 0.00 aB
EC4	0.13 ± 0.01 abA	0.18 ± 0.01 abcAB	0.20 ± 0.01 aAB	0.23± 0.02 aB
EC5	0.13 ± 0.00 aA	0.17 ± 0.00 abcB	0.17 ± 0.00 aB	0.23 ± 0.00 aC
EC6	0.11 ± 0.00 aA	0.17 ± 0.00 abcB	0.20 ± 0.00 aC	0.25 ± 0.00 aD
EC7	0.11 ± 0.01 aA	0.16 ± 0.01 abAB	0.19 ± 0.00 aBC	0.22 ± 0.01 aC
	Mean lipid particle size (µm)			
EP	2.05 ± 0.07 abA	2.10 ± 0.03 abA	2.12 ± 0.06 aA	2.13 ± 0.12 aA
EC3	1.92 ± 0.10 aA	1.96 ± 0.11 aA	2.05 ± 0.02 aA	2.07 ± 0.04 aA
EC4	1.97 ± 0.08 aA	2.01 ± 0.09 aA	2.03 ± 0.01 aA	2.07 ± 0.01 aA
EC5	2.02 ± 0.04 abA	2.05 ± 0.04 aA	2.05 ± 0.04 aA	2.12 ± 0.05 aA
EC6	2.03 ± 0.05 abA	2.12 ± 0.04 abA	2.16 ± 0.05 aA	2.15 ± 0.04 aA
EC7	2.01 ± 0.03 abA	2.19 ± 0.04 abAB	2.20 ± 0.05 aAB	2.26 ± 0.07 abB

For denomination of cream-type emulsions, see Figure 1. Mean values ± standard deviation of the parameter with different lower-case letters in the same column are significantly different (grouping variable: cream type), *p*-value < 0.05; Mean values ± standard deviation of the parameter with different uppercase letters in the same row are significantly different (grouping variable: NaCl concentration), *p*-value < 0.05.

## Data Availability

The original contributions presented in the study are included in the article/Supplementary Materials, further inquiries can be directed to the corresponding author.

## Supplementary Materials

The following supporting information can be downloaded at: https://www.mdpi.com/article/10.3390/foods13050729/s1.
