# Supplementary material for: New Insight into Food-Grade Emulsions: Candelilla Wax-Based Oleogels as an Internal Phase of Novel Vegan Creams
Source: Foods. 2024 Feb 28;13(5):729. doi: 10.3390/foods13050729 (PMC10930791; doi:10.3390/foods13050729)
Supplement: Supplementary file 1 [file foods-13-00729-s001.zip › foods-2865982-supplementary.pdf]

SUPPLEMENTARY METARIAL:

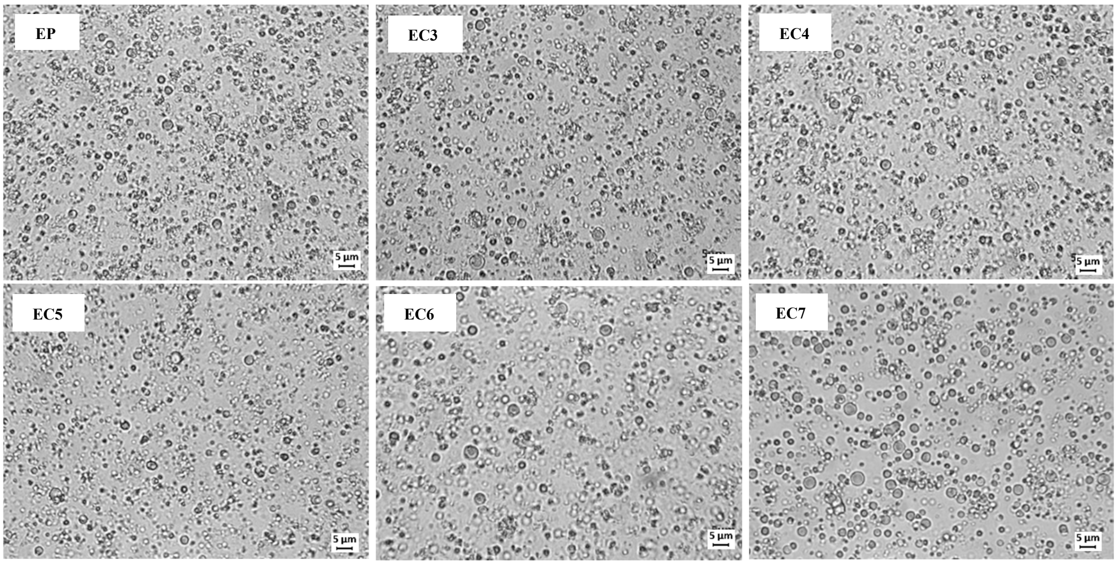

Figure S1. Micrographs of fresh (24 h after production) cream-type emulsions in 600x magnification (EP – cream-type emulsions based on palm oil; EC3/4/5/6/7 – cream-type emulsions based on oleogel with 3, 4, 5, 6 or 7% w/w candelilla wax.)

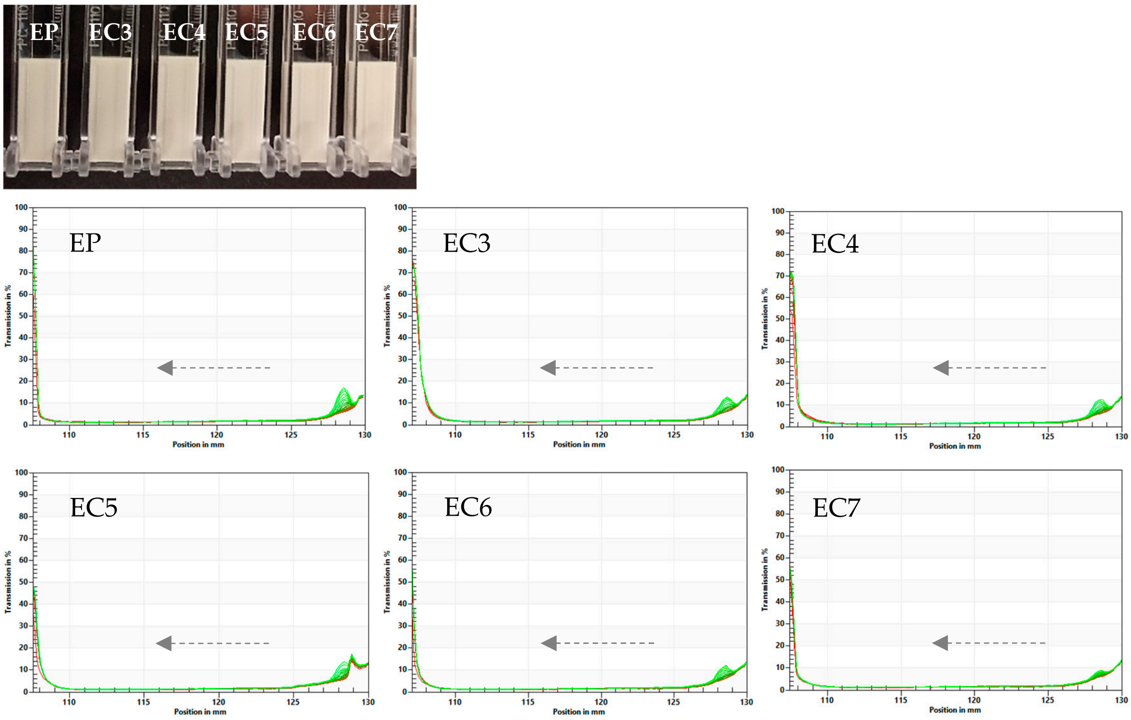

Figure S2. Transmission profiles of "fresh" (24 h after production) cream-type emulsions
For denomination of cream-type emulsions, see below Fig. S1.
Position 0 mm – the bottom of the sample, position 130 mm – the meniscus of the sample;
Grey arrows in the graphs indicate the direction of movement of the particles during centrifugation.

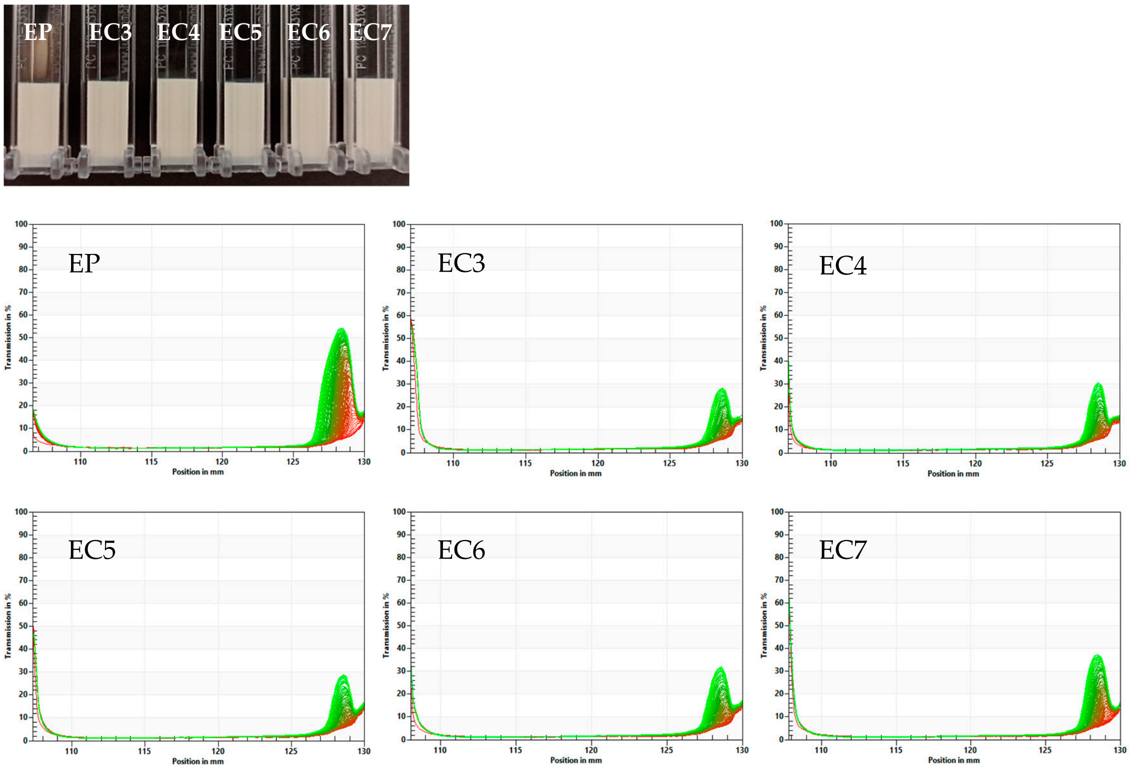

Figure S3. Transmission profiles of cream-type emulsions after heating at 90°C for 30 min

For denomination of cream-type emulsions, see below Fig. S1.

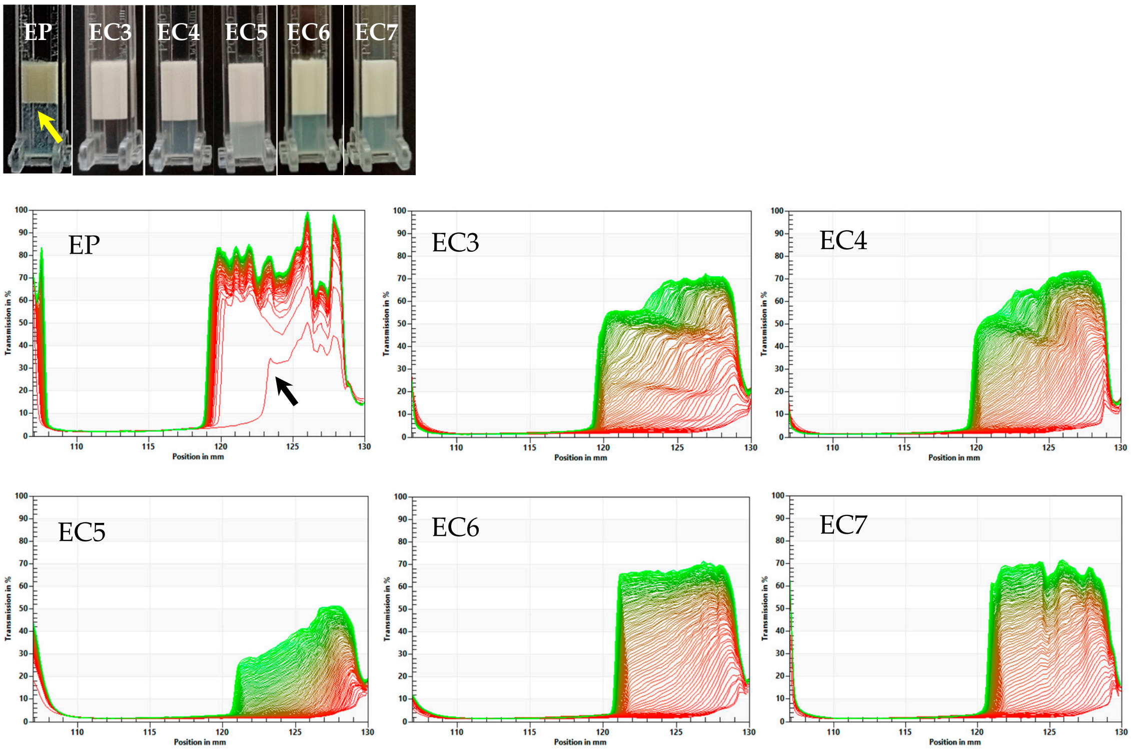

Figure S4. Transmission profiles of cream-type emulsions after freeze-thaw cycle

For denomination of cream-type emulsions, see below Fig. S1.

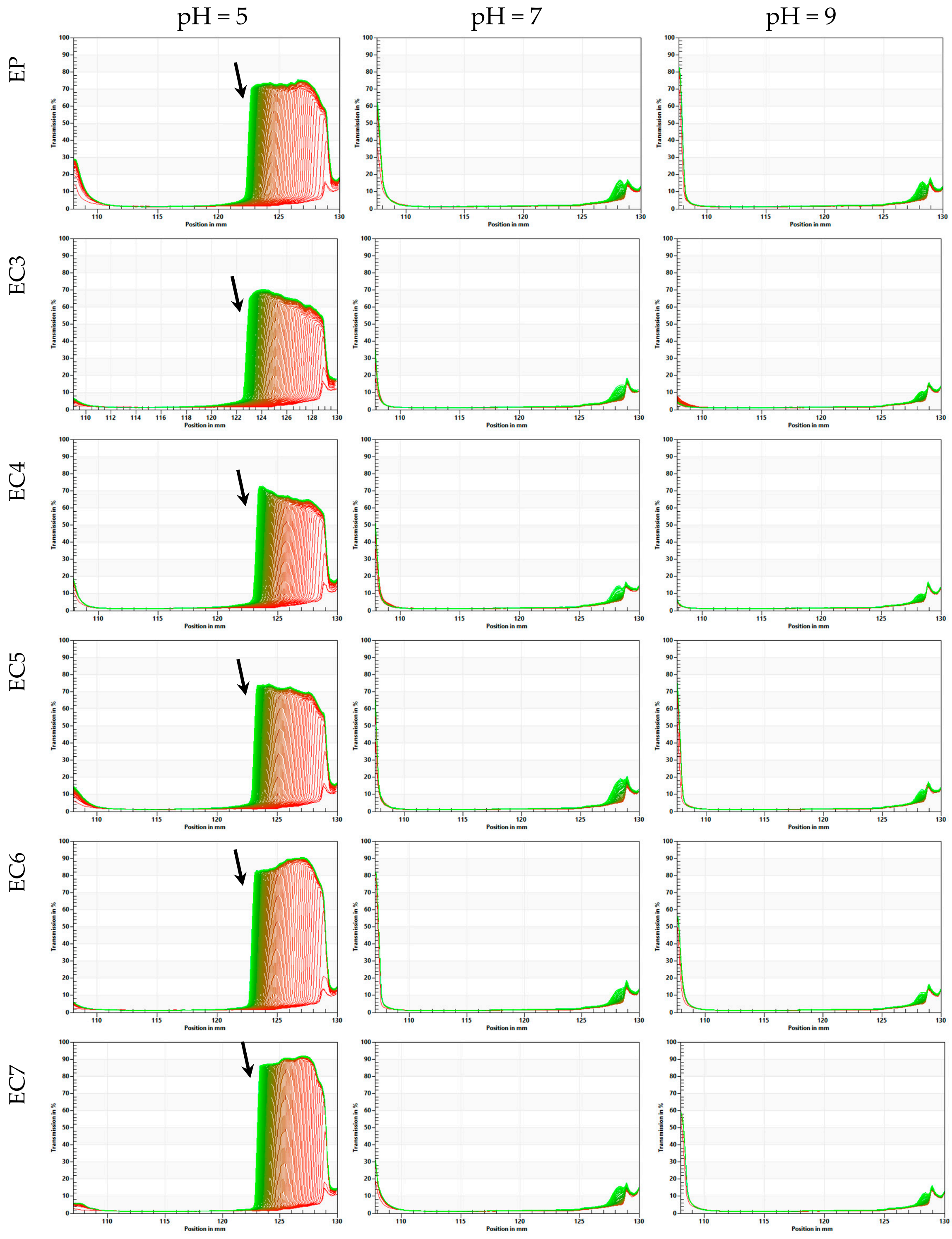

Figure S5. Transmission profiles of cream-type emulsions dispersed in solutions with a pH of 5, 7 or 9 (1:1)

For denomination of cream-type emulsions, see below Fig. S1.

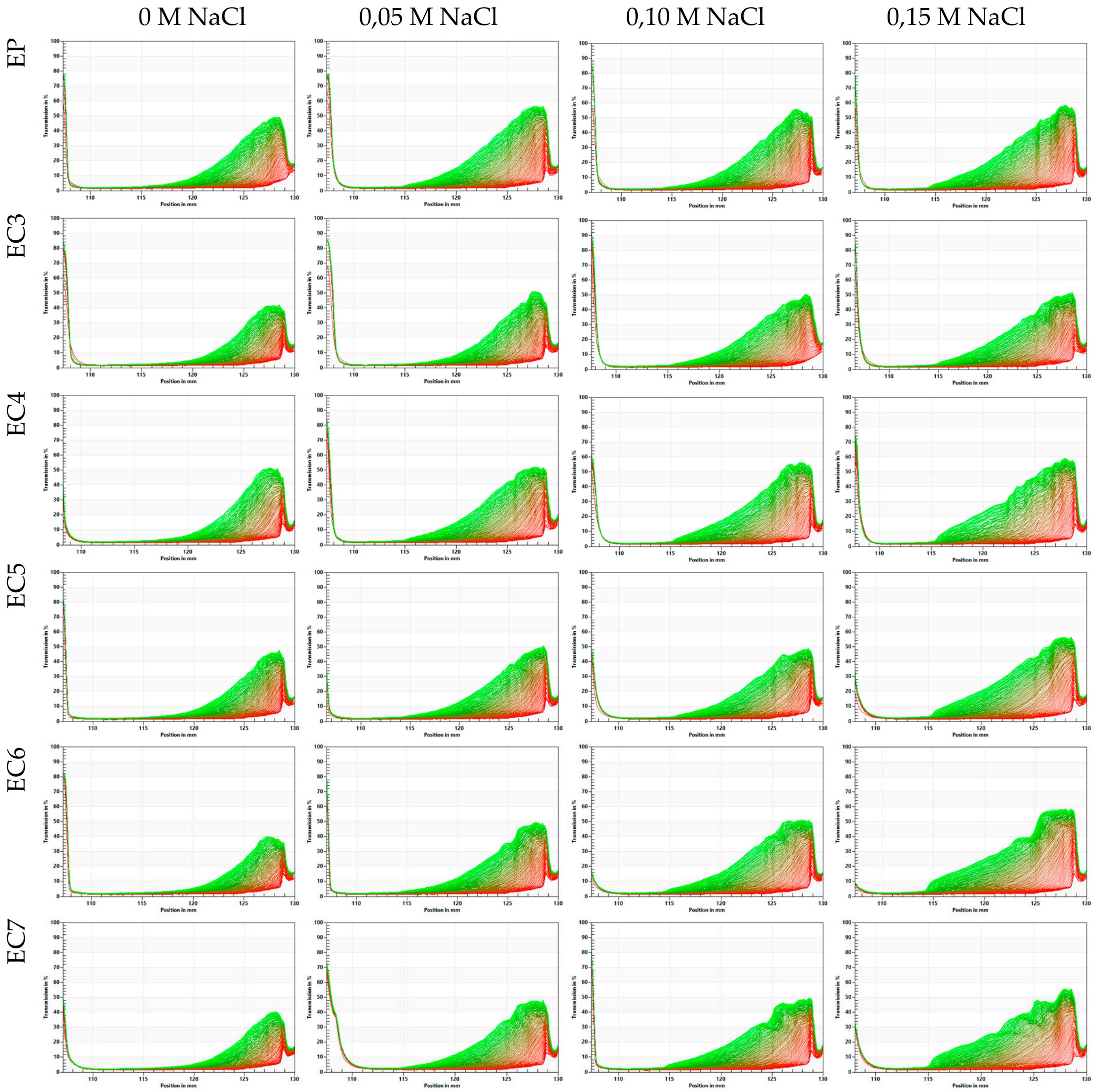

Figure S6. Transmission profiles of cream-type emulsions dispersed in solutions with a NaCl concentration 0 M, 0.05 M, 0.1 M or 0.15 M (1:1)

For denomination of cream-type emulsions, see below Fig. S1.
